# The regulations on cortical activation and functional connectivity of the dorsolateral prefrontal cortex-primary somatosensory cortex elicited by acupuncture with reinforcing-reducing manipulation

**DOI:** 10.3389/fnhum.2023.1159378

**Published:** 2023-05-03

**Authors:** Jingya Cao, Yuzhu Qu, Li Chen, Tianyu Liu, Jing Guo, Yulai Gong, Zilei Tian, Jing Xiong, Zhenfang Lin, Xin Yang, Tao Yin, Fang Zeng

**Affiliations:** ^1^Acupuncture and Tuina School, Chengdu University of Traditional Chinese Medicine, Chengdu, Sichuan, China; ^2^Acupuncture and Brain Science Research Center, Chengdu University of Traditional Chinese Medicine, Chengdu, Sichuan, China; ^3^Sport and Healthy School, Chengdu University of Traditional Chinese Medicine, Chengdu, Sichuan, China; ^4^Department of Neurology, Sichuan Provincial Rehabilitation Hospital, Chengdu, Sichuan, China; ^5^Rehabilitation Medicine Center and Institute of Rehabilitation Medicine, West China Hospital, Sichuan University, Chengdu, Sichuan, China; ^6^Health and Rehabilitation School, Chengdu University of Traditional Chinese Medicine, Chengdu, Sichuan, China

**Keywords:** neuroimaging, central mechanism, functional near-infrared spectroscopy, acupuncture, reinforcing-reducing manipulation

## Abstract

**Introduction:**

Traditional acupuncture with reinforcing-reducing manipulation is essential for clinical effectiveness, whereas the underlying central mechanism of it remains unknown. This study with multiple-channels functional near-infrared spectroscopy (fNIRS) aims to explore cerebral-response modes during acupuncture with reinforcing-reducing manipulations.

**Materials and methods:**

Functional near-infrared spectroscopy data were recorded from 35 healthy participants during the lifting-thrusting reinforcing manipulation, the lifting-thrusting reducing manipulation, and the even reinforcing-reducing manipulation with lifting-thrusting. The general linear model based (GLM) cortical activation analysis and the functional connectivity (FC) based on region of interest (ROI) analysis were combined to be conducted.

**Results:**

In comparison with the baseline, the results showed that three acupuncture with reinforcing-reducing manipulations similarly induced the hemodynamic responses in the bilateral dorsolateral prefrontal cortex (DLPFC) and increased FC between the DLPFC and primary somatosensory cortex (S1). Specifically, the even reinforcing-reducing manipulation deactivated the bilateral DLPFC, the frontopolar area (FP), the right primary motor cortex (M1), the bilateral S1, and the bilateral secondary somatosensory cortex (S2); The reducing manipulation deactivated the bilateral DLPFC; The reinforcing manipulation activated the bilateral DLPFC, the left S1, and the right S2. The between-group comparisons indicated that the reinforcing-reducing manipulation induced opposite hemodynamic responses in the bilateral DLPFC and the left S1 and exhibited different FC patterns in the left DLPFC-S1, within the right DLPFC, and between the left S1 and the left orbitofrontal cortex (OFC).

**Conclusion:**

These findings verified the feasibility of fNIRS for investigating cerebral functional activities of acupuncture manipulations, suggesting that the regulations on the DLPFC-S1 cortex may be the potential central mechanism for the realization of acupuncture with reinforcing-reducing manipulation’s effect.

**Clinical trial registration:**

ClinicalTrials.gov, identifier, ChiCTR2100051893.

## 1. Introduction

Acupuncture has been widely used all over the world due to its efficacy for many diseases (Liu et al., 2021; Lu et al., 2022). According to ancient books, modern research, and clinical practice, acupuncture with reinforcing-reducing manipulation is key to clinical efficacy (Shi et al., 2012). The essential role of acupuncture manipulation in acupuncture’s efficacy has been proven in numerous clinical studies. For instance, acupuncture manipulation had better efficacy than acupuncture without manipulation for Bell’s palsy patients (Uchino, 2013). The reinforcing manipulation is superior to even reinforcing-reducing and reducing manipulation for improving muscle force (Du et al., 2016), increasing cerebral blood flow, and lowering the infarct volume ratio in patients with acute ischemic stroke (Zhang et al., 2013).

Among multiple acupuncture with reinforcing-reducing manipulations, lifting-thrusting reinforcing-reducing manipulations are most commonly applied in the clinic, as the foundation for complex acupuncture with reinforcing-reducing manipulations (Wu et al., 2019). The even reinforcing-reducing manipulation with lifting-thrusting (ERR) is usually used for clinical treatment and treating for complicated excessiveness and deficiency syndromes or balanced excessiveness and deficiency syndromes by lifting and thrusting with even force and amplitude. The lifting-thrusting reinforcing manipulation (LTRei) is used for strengthening the body’s resistance and treating deficiency syndromes by thrusting heavily and lifting gently with a low frequency (Wu et al., 2019). The lifting-thrusting reducing manipulation (LTRed) is adopted for eliminating the pathogenic factors and relieving excess syndrome by lifting heavily and thrusting gently with a high frequency (Wu et al., 2019).

Central integration is important for acupuncture. Using neuroimaging technologies, a non-invasive visualization method, studies on the central mechanism of acupuncture manipulation have been conducted. For example, a positron emission computed tomography (PET) study explored the central mechanism that how the reinforcing-reducing manipulation works for the decrease of blood pressure (Guo et al., 2018). Another functional magnetic resonance imaging (fMRI) study investigated the effects of three acupuncture manipulations (twirling, lifting-thrusting, and twirling plus lifting-thrusting) on brain functional activities and found that these acupuncture manipulations also activated areas associated with the cognition, sensory, vision, and emotional regulation, which might be the potential targets for acupuncture manipulations’ efficacy (Lu et al., 2017). However, the central mechanism of acupuncture manipulations has remained unclear so far mainly due to the following two problems. Firstly, due to the low temporal resolution of fMRI and PET, the changes in functional cerebral activities during acupuncture manipulations cannot be monitored within a short time. Secondly, both fMRI and PET were conducted in a strict laboratory situation, the outcomes might not be generalized in a real clinical situation.

To address the above two issues, functional near-infrared spectroscopy (fNIRS) was adopted in the current study. Similar to fMRI, fNIRS detects the cerebral cortex’s hemodynamic responses based on the neurovascular coupling effect (Soltanlou et al., 2018). In comparison with PET and fMRI, fNIRS with a higher temporal resolution (Chen et al., 2020) could quickly and accurately monitor functional cerebral activities of acupuncture manipulations in a real clinical setting. Compared to electroencephalogram (EEG), fNIRS with a higher spatial resolution (Chen et al., 2020) might measure metabolic responses (Putze et al., 2014) to acupuncture manipulations with more sensitive localized activation (Wu et al., 2018). Using fNIRS, a study detected the existence of different cortical networks of acupuncture manipulation and the anesthetic effect of the *Hegu* (LI4).

Therefore, the multi-channel fNIRS was applied to record the cortical hemodynamic response of acupuncture recipients during ERR, LTRei, and LTRed. Then, the within-group analysis was performed to clarify the brain response patterns of these three manipulations, and the between-group comparison was conducted to investigate the differences in brain responses among the three modes of manipulations. This study hypothesized that ERR, LTRei, and LTRed would have different patterns of cortical responses, manifesting as cortical activity and functional brain connectivity.

## 2. Materials and methods

### 2.1. Participants

A total of 35 healthy participants were recruited from the Chengdu University of Traditional Chinese Medicine (CDUTCM). Participants were included if they fulfilled the following criteria: (1) right-handed male between the ages of 18 and 40, and (2) with a college degree or higher, and (3) having none of the organic or functional diseases in each system or mental disorders, and (4) having not taken part in any other clinical research in the last 3 months, and (5) given informed consent. Participants were excluded: (1) having severe skin disorders, infections, or damaged skin at acupoints, or (2) having a severe needle phobia (headache, dizziness, cold sweat, chest tightness, etc.) or (3) having a score of 50 or above on the Self-rating Depression Scale (SDS) or Self-rating Anxiety Scale (SAS) (Zung, 1971).

The enrolled participants received ERR, LTRei, and LTRed in a randomized order, each was separated by a 1 day washout (Bai et al., 2009; Hui et al., 2012). The order of performing acupuncture manipulations was generated by a randomization digital table.

This study was performed according to the principles of the Declaration of Helsinki. The study protocol has been approved by the Institutional Review Board of the Hospital of Chengdu University of Traditional Chinese Medicine (approved number: 2021QKL-001).

### 2.2. Acupuncture manipulation

**FIGURE 1:**
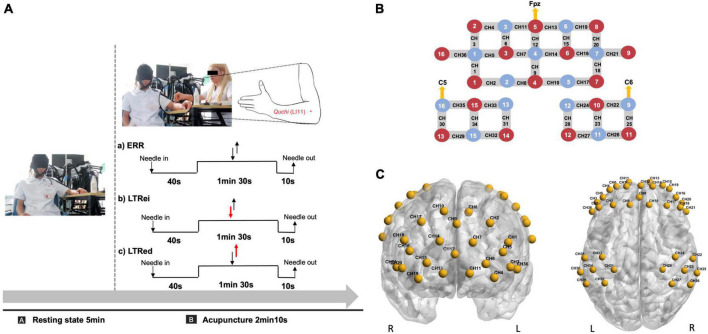
**(A)** Acupuncture and functional near-infrared spectroscopy (fNIRS) scan diagram. The duration of acupuncture manipulation lasted for 1 min 30 s. Two reverse black arrows indicate that the lift-thrust is conducted with even force and speed. The red-down arrow and black-up arrow indicate that the needle is thrusted heavily and lifted lightly. The red-up arrow and black-down arrow indicate that the needle is lifted heavily and thrusted lightly. **(B,C)** Optode and channel configuration. **(B)** An optode probe set with 16 sources and 13 detectors. The red dot represents the source, the blue dot represents the detector, and the line between them represents the channel. **(C)** The channels were positioned on the bilateral prefrontal and bilateral somatosensory cortex. CH, channel.

The same licensed acupuncturist with over 5 years of practical experience performed all acupuncture manipulations at *Quchi* (LI11, left side), using disposable sterile needles (0.25 × 40 mm, Suzhou Huatuo Medical Instrument Co., Ltd., China). The flow diagram of acupuncture is shown in Figure 1A and the details of each acupuncture manipulation were as follows.

Before each acupuncture manipulation, the needle was perpendicularly inserted at a depth of 15 mm, thrusted, and lifted with even force and speed to elicit the needling sensation.

(1) LTRei: Conducted with heavy thrust and gentle lift, a lower frequency (<1 Hz), and an amplitude of 3∼5 mm.

(2) LTRed: Performed with heavy lift and gentle thrust, a higher frequency (>1.5 Hz), and an amplitude of 3∼5 mm.

(3) ERR: Lifted and thrusted with even force and speed with a frequency of 1∼1.5 Hz and an amplitude of 3∼5 mm.

### 2.3. Clinical variables assessments

After each acupuncture manipulation, the needling sensation was evaluated by the Chinese version of the modified Massachusetts General Hospital Acupuncture Sensation Scale (C-MMASS) (Yu et al., 2012). In the form, the 10-point Visual Analogue Scale (VAS) scale was used to assess seven categories of needling sensation including soreness, numbness, fullness, heaviness, aching, throbbing, and other sensations, respectively.

The Mindful Attention Awareness Scale (MAAS) (Brown and Ryan, 2003) was used to assess participants’ current attention and awareness. The SAS (Zung, 1971) and SDS (Zung, 1965) were used to evaluate the emotional wellbeing of participants. These assessments were administered at baseline.

### 2.4. fNIRS data acquisition

#### 2.4.1. Optode and channel configuration

Functional near-infrared spectroscopy data were collected at 3.096 Hz using a continuous wave (CW) instrument (NIRx Medical Technologies, NY, USA). The concentration signals of oxygenated hemoglobin (HbO) and deoxygenated hemoglobin (HbR) were detected using two different wavelengths of near-infrared light (785 and 830 nm).

For the optode configuration, 16 sources and 13 detectors were set on the bilateral prefrontal cortex (PFC) and bilateral somatosensory cortex, which referred to the standard international 10–10 system of electrode placement (Jurcak et al., 2007). The lowest middle optode of the PFC (source number 5) and the outer optode of the bilateral somatosensory cortex (detector numbers 16 and 9) were located at Fpz, C5, and C6, respectively. The distance between each source and detector pair was approximately 3 cm, as shown in Figure 1B.

The 36-channel Montreal Neurological Institute (MNI) coordinates were transformed from the spatial coordinates of the midpoint between each pair of sources and detectors by the NIRS-SPM toolbox and then were visualized by BrainNet Viewer toolbox (Xia et al., 2013; Figure 1C). The details of the MNI coordinates of each channel and their corresponding anatomical location are shown in Supplementary Table 1.

#### 2.4.2. fNIRS scan paradigm

functional near-infrared spectroscopy scans for 7 min 15 s were performed simultaneously with acupuncture manipulations. Following a 5 min resting-state fNIRS scan, the needle was inserted, followed by lifting and thrusting to induce the needling sensation. Then acupuncture manipulations were performed for 1 min 30 s. Participants were instructed to stay awake, keep their heads still, and pay attention to the needling sensation throughout the whole scan. The paradigm of the fNIRS scan is shown in Figure 1A.

### 2.5. Statistical analysis

#### 2.5.1. Sample size

Although there is no standard for sample-size calculation in acupuncture-neuroimaging studies (Qiu et al., 2016; Fede et al., 2020), 12∼16 participants per group have been usually used (Hayasaka et al., 2007; Qiu et al., 2016; Murase et al., 2021). Larger sample sizes have been recommended to achieve stable statistical power and repeatable results (at least 20 participants per group) (Chen et al., 2018; Fede et al., 2020). Therefore, 30 participants were planned to be recruited. Considering a 15% dropout rate, 35 participants were finally enrolled (Desmond and Glover, 2002).

#### 2.5.2. Needling sensation analysis

One-way repeated-measures analysis of variances (One-way rmANOVAs) were selected for between-group comparisons. The level of statistical significance was *p* < 0.05, and a two-tailed test was used.

#### 2.5.3. fNIRS data analysis

##### 2.5.3.1. Data preprocessing

Functional near-infrared spectroscopy data preprocessing was performed with Homer2 toolbox based on MATLAB (R2013b, MathWorks, Inc., Natick, MA, USA). The preprocessing procedures were conducted as follows: (1) Optical density (OD) was transferred from optical intensity using the function *hmrintensity2OD*; (2) The channels with too many motion artifacts were rejected by the function *hmrMotionArtifactByChannel*; (3) Motion correction was conducted by the function *hmrMotionCorrectSpline*; (4) The physiological noises such as cardiac (1–2 Hz), respiration (0.2–0.4 Hz), etc., were removed by the function *hmrBandpassFilt* (0.01 < f < 0.1 Hz) (Pinti et al., 2019); and (5) The OD was converted into hemoglobin concentration changes using the function *hmrOD2Conc.*

##### 2.5.3.2. Cortical activation analysis

After the preprocessing was completed, fNIRS data based on HbO level was selected for further analysis due to HbO is the most sensitive index for measuring changes in blood flow (Hoshi et al., 2001). Based on a general linear model (GLM) approach, the cortical activation during acupuncture manipulations were calculated (Pinti et al., 2017). The regression coefficient (β-value), one of the GLM’s weights (Hou et al., 2021), was used to indicate the changes in cerebral hemodynamic responses during acupuncture manipulation in the current study. β-values for 36 channels were calculated by the NIRS-SPM.

To investigate the cortical activation during each reinforcing-reducing manipulation, paired *t*-tests were selected for within-group comparisons. To investigate the differences in cortical activation among three reinforcing-reducing manipulations, One-way rmANOVAs were selected for between-group comparisons, and the channels with significant differences were regarded as the regions-of-interest (ROIs) for subsequent task-state functional connectivity (FC) analysis.

##### 2.5.3.3. Functional connectivity analysis

*Pearson’s* correlation coefficients were used to characterize FC between ROIs and other channels (Novi et al., 2016). For yielding variants from the normal distribution, correlation values were converted to Fisher *z*-values by Fisher *z*-transformation (Ghafoor et al., 2019).

To investigate alternations in FC during each reinforcing-reducing manipulation, paired *t*-tests were selected for within-group comparison. To investigate the differences in FC among three reinforcing-reducing manipulations, One-way rmANOVAs were selected for between-group comparisons.

In this study, all multiple comparisons for brain regions were performed by the false discovered rates method (FDR, *p* < 0.05) (Glickman et al., 2014). Multiple comparisons for *post-hoc* tests were performed by *Bonferroni* corrections. The level of statistical significance was *p* < 0.05, and a two-tailed test was used.

## 3. Results

**FIGURE 2:**
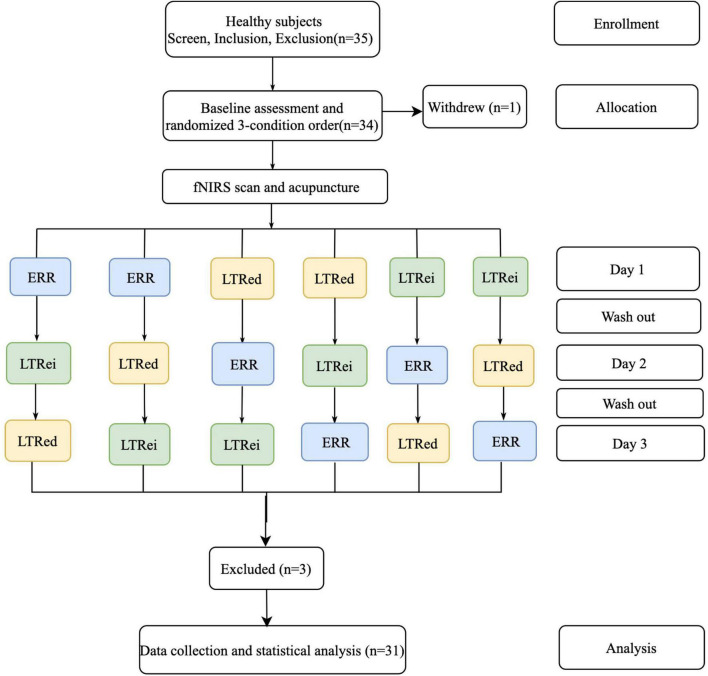
The flow chart of research design and participant allocation. ERR, even reinforcing-reducing manipulation with lifting-thrusting; LTRei, lifting-thrusting reinforcing manipulation; LTRed, lifting-thrusting reducing manipulation; fNIRS, functional near-infrared spectroscopy.

Among total of 35 participants who were originally involved in this study, one participant withdrew due to time conflict, and three-participant fNIRS data had file defects. Thus, the remaining 31-participant data were analyzed. The flow chart of this study is shown in Figure 2.

### 3.1. Baseline information

A total of 31-participant baseline information including age, body mass index (BMI), emotional condition, and cognitive condition is shown in Table 1.

**TABLE 1:** Baseline information.

Characteristic	mean ± SD
Age (years)	20.70 ± 2.34
BMI (kg/m^2^)	22.02 ± 3.37
MAAS (points)	69.41 ± 8.23
SAS (points)	32.70 ± 8.40
SDS (points)	34.11 ± 10.04

SD, standard deviation; BMI, body mass index; MAAS, The Mindful Attention Awareness Scale; SAS, The Self-rating Anxiety Scale; SDS, The Self-rating Depression Scale.

### 3.2. Comparison of the needling sensation

**FIGURE 3:**
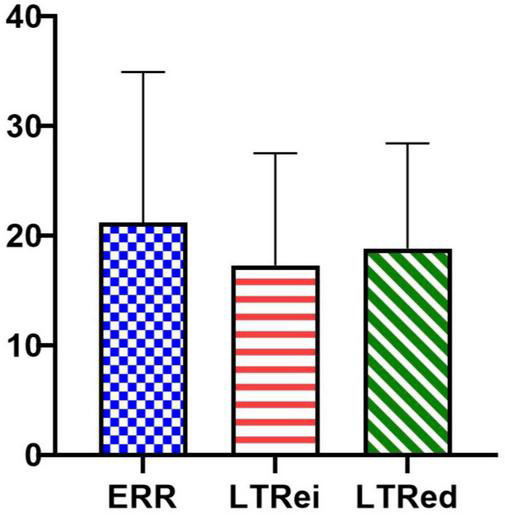
The comparison of the needling sensation among three acupuncture manipulations.

The comparison of the needling sensation showed no statistically significant differences among ERR, LTRei, and LTRed [Figure 3, *F*_(2,60)_ = 2.917, *p* = 0.062, η^2^ = 0.089].

### 3.3. Cortical activation during each reinforcing-reducing manipulation

**FIGURE 4:**
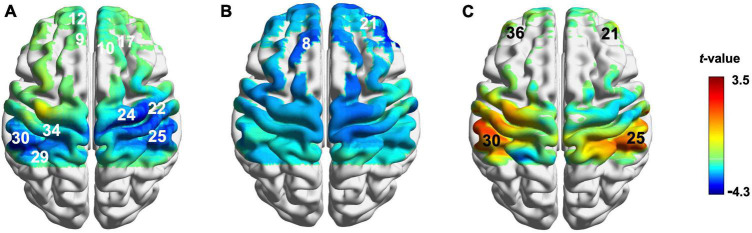
Cortical activation during reinforcing-reducing manipulations. **(A)** Even reinforcing-reducing manipulation with lifting-thrusting; **(B)** lifting-thrusting reducing manipulation; **(C)** lifting-thrusting reinforcing manipulation. The number (channel) in white represents decreased hemodynamic responses and the number (channel) in black represents increased hemodynamic responses.

Compared with the baseline, ERR induced a decrease of hemodynamic responses in the bilateral DLPFC, the left frontopolar area (FP), the right primary motor cortex (M1), the bilateral primary somatosensory cortex (S1), and the bilateral secondary somatosensory cortex (S2) (Figure 4A and Supplementary Table 2); LTRed elicited a decrease of hemodynamic responses in the bilateral DLPFC (Figure 4B and Supplementary Table 2); LTRei induced an increase of hemodynamic responses in the bilateral DLPFC, the left S1, and the right S2 (Figure 4C and Supplementary Table 2).

### 3.4. Comparisons of cortical activation among three reinforcing-reducing manipulations

**FIGURE 5:**
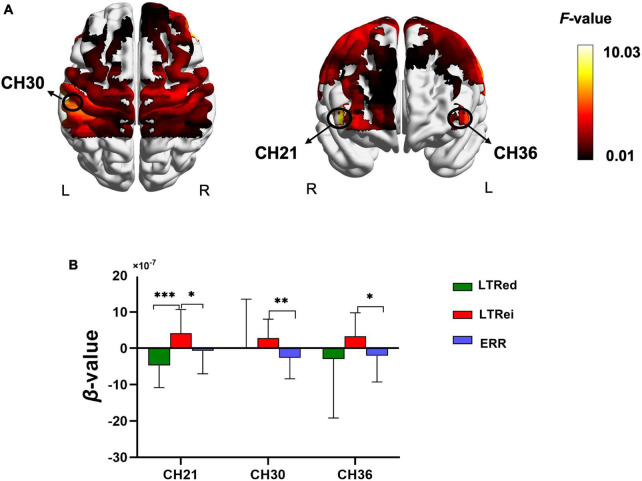
The comparison of cortical activation among three acupuncture manipulations. **(A)** The cerebral activation with significant differences (Channel number 21, 30, and 36) among three acupuncture manipulations. **(B)** *Post-hoc* tests for β-values. ^***^p < 0.001, ^**^p < 0.01, *p < 0.05.

The comparison of cortical activation among three reinforcing-reducing manipulations showed significant differences in the bilateral DLPFC [CH21, *F*_(2,58)_ = 10.029, *p* < 0.001, η^2^ = 0.257; CH36, *F*_(2,28)_ = 4.746, *p* = 0.017, η^2^ = 0.253] and left S1 [CH30, *F*_(2,28)_ = 6.56, *p* = 0.005, η^2^ = 0.319] (Figure 5A). Compared to ERR, LTRei elicited an increase of hemodynamic responses in the bilateral DLPFC and left S1 (*p* = 0.031, 0.027, and 0.004, respectively), while LTRed showed no statistically significant differences (Figure 4B). The comparison between LTRei and LTRed demonstrated opposite hemodynamic responses in the right DLPFC (*p* < 0.001) (Figure 5B).

### 3.5. Functional connectivity during each reinforcing-reducing manipulation

The bilateral DLPFC (CH21 and CH36) and the left S1 (CH30) with significant differences in cortical activation analysis among reinforcing-reducing manipulations were selected as ROIs to explore the brain network characteristics of reinforcing-reducing manipulations.

**FIGURE 6:**
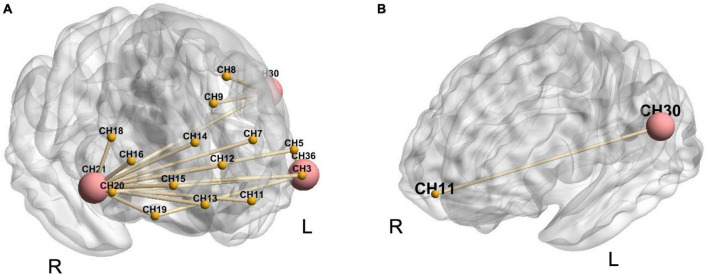
The alternations in functional connectivity between region of interest (ROIs) and other 36 channels during each reinforcing-reducing manipulation. The big-red node is ROI, the small-yellow node is the other channel, and the yellow line between nodes represents the edge with significant differences. **(A)** Lifting-thrusting reinforcing manipulation; **(B)** lifting-thrusting reducing manipulation.

Compared to the baseline, all reinforcing-reducing manipulations showed increased FC. LTRei significantly showed increased FC within the right DLPFC (CH21-16, *t* = 3.17, *p* = 0.023; CH21-18, *t* = 3.29, *p* = 0.022; CH21-20, *t* = 4.74, *p* = 0.005), between the bilateral DLPFC (CH21-3, *t* = 2.79, *p* = 0.044; CH21-5, *t* = 3.15, *p* = 0.023; CH21-36, *t* = 3.25, *p* = 0.022; CH20-36, *t* = 2.86, *p* = 0.039), between the right DLPFC and bilateral FP (CH21-7, *t* = 3.01, *p* = 0.031; CH21-11, *t* = 3.57, *p* = 0.014; CH21-12, *t* = 3.59, *p* = 0.014; CH21-13, *t* = 3.62 *p* = 0.014; CH21-14, *t* = 2.94, *p* = 0.035; CH21-15, *t* = 3.42, *p* = 0.018), between the right orbitofrontal cortex (OFC) and bilateral DLPFC (CH19-21, *t* = 4.13, *p* = 0.014; CH19-36, *t* = 3.20, *p* = 0.023), and between the left S1 and bilateral DLPFC (CH30-21, *t* = 3.53, *p* = 0.014; CH30-36, *t* = 3.53, *p* = 0.014) (Figure 6A). LTRed significantly demonstrated increased FC between the left S1 and left OFC (CH30-11, *t* = 4.55, *p* = 0.009) (Figure 6B). However, ERR showed no statistically significant differences.

### 3.6. Comparisons of functional connectivity among three reinforcing-reducing manipulations

**FIGURE 7:**
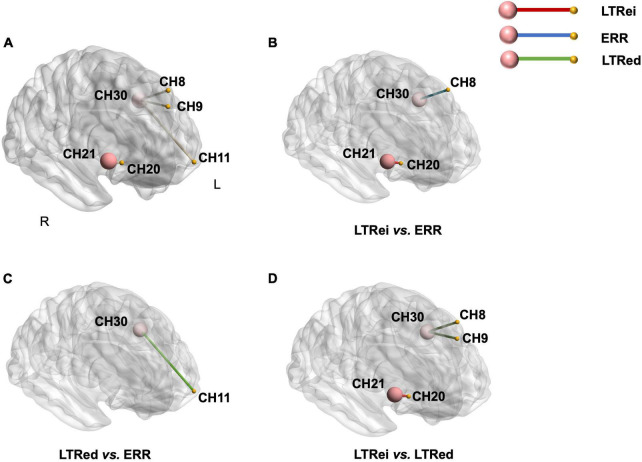
A comparison of functional connectivity among three reinforcing-reducing manipulations. The big-red node is region of interest (ROI), the small-yellow node is the other channel. **(A)** The functional connectivity (the yellow edge between nodes) with significant differences among three acupuncture manipulations; **(B–D)** *post-hoc* tests for functional connectivity. The edge with color indicates that functional connectivity strength is stronger than the other.

The comparison of FC among three reinforcing-reducing manipulations showed significant differences within the right DLPFC [CH21-20, *F*_(2,58)_ = 5.881, *p* = 0.005, η^2^ = 0.169], between the left S1 and left DLPFC [CH30-8, *F*_(2,58)_ = 7.680, *p* = 0.001, η^2^ = 0.209; CH30-9, *F*_(2,58)_ = 5.311 *p* = 0.008, η^2^ = 0.155], and between the left S1 and left OFC [CH30-11, *F*_(2,58)_ = 3.868, *p* = 0.026, η^2^ = 0.118] (Figure 7A). Compared to ERR, LTRei showed stronger FC within the right DLPFC (CH21-20, *p* = 0.004) and LTRed showed stronger FC between the left S1 and left OFC (CH30-11, *p* = 0.046) (Figures 7B, C). In comparison with LTRei, LTRed indicated stronger FC strength between the left S1 and left DLPFC (CH30-8, *p* = 0.002; CH30-9, *p* = 0.004) (Figure 7D).

## 4. Discussion

This study verified the feasibility of fNIRS for investigating the cortical-response modes of different reinforcing-reducing manipulations and found that three reinforcing-reducing manipulations all induced cerebral hemodynamic responses in the bilateral DLPFC and increased functional connectivity in DLPFC-S1. The comparison of cerebral functional activities showed opposite hemodynamic responses in the bilateral DLPFC and the left S1, and different functional connectivity patterns in the left DLPFC-S1, within the right DLPFC, and the left S1-OFC among three reinforcing-reducing manipulations.

Massive acupuncture-neuroimaging studies have been conducted in the past two decades. As an acupuncture therapy, three reinforcing-reducing manipulations all induced changes in cerebral functional activities in the bilateral DLPFC, which is similar to previous studies (Cho et al., 2013; Lu et al., 2017). Meanwhile, increased synchronization of brain regions was induced by all acupuncture manipulations, which indicate that the reinforcing-reducing manipulation could enhance the information transmission through the sensory gating system consisting of DLPFC and S1 (Staines et al., 2002; Gogulski et al., 2015). In recent years, the central mechanism of acupuncture manipulations has been increasingly investigated. For example, an fMRI study showed that the activated signals induced by the lifting-thrusting manipulation were stronger than other acupuncture manipulations (Lu et al., 2017). Similarly, the between-group comparison of cortical activation showed that the reinforcing manipulation induced an increased signal, while the reducing manipulation elicited a decreased signal in the bilateral DLPFC and the left S1. Interestingly, the comparison of the needling sensation among three reinforcing-reducing manipulations showed no statistically significant differences, which is different from the previous study’s results (Hori et al., 2010).

The mechanism of acupuncture’s efficacy through the pathway from the periphery to the central nervous system has been increasingly investigated (Napadow et al., 2020). The neurologic model and the connective tissue model are the two dominant models for the initiation of acupuncture signals. When performing lifting-thrusting reinforcing-reducing manipulations, different afferent nerve fibers in different layers of the muscle tissue are involved (Lu et al., 2021; Guo et al., 2022) and different types of peripheral sensory afferents are activated, which are essential in distinguishing sensory stimuli (Prsa et al., 2019). A study found a link between regional collagen fibers at acupoints and the efficacy of acupuncture manipulations, as well as that the fibrillar collagen’s response at LI11 to the needle’s lift-thrust varies with tissue stiffness and density and the depth of needle insertion (Wang et al., 2017). The afferent signals are transmitted via the somatosensory pathway in the spinal dorsal column (Chang et al., 2017) and the A-fibers of the ulnar nerve (Kim et al., 2013) during acupuncture manipulations. Thus, this study speculates that different cortical responses might be attributed to different acupuncture-manipulation modes (e.g., frequency, intensity, amplitude, and direction).

As the predominant somatosensory cortex, the S1 is particularly sensitive to the frequency of stimuli (Kalberlah et al., 2013) and processes multiple sensory factors influencing the perception of somatosensory stimuli such as the intensity (Chen et al., 2002), location (Beauchamp et al., 2009), and modality (Yang et al., 2018). As the key node of PFC (Staines et al., 2002), the DLPFC plays an important role in sensory discrimination (Pleger et al., 2006; Kostopoulos et al., 2007), perceptual decision-making (Staines et al., 2002; Heekeren et al., 2008), and emotional processing (Seminowicz and Moayedi, 2017). According to traditional Chinese medicine, the differences in frequency, intensity, amplitude, and direction among reinforcing-reducing manipulations (Shi, 2021) might lead to different cerebral responses in the DLPFC and S1. Through the PFC-dominant central nervous pathway, the dynamic balance of sympathetic and parasympathetic nerves within autonomic nerves system (ANS) is regulated by acupuncture (Wang et al., 2002; Chi et al., 2018). A review demonstrated that the regulation on ANS by acupuncture is one of the most crucial mechanisms for acupuncture’s efficacy (Li et al., 2022) and the changes within ANS could be characterized by hemodynamic responses in brain regions (Hori et al., 2010). Therefore, this study speculates that the regulation on the DLPFC-S1 might be the potential central mechanism of the realization of the reinforcing-reducing manipulations’ effect.

Functional near-infrared spectroscopy has been applied in acupuncture-neuroimaging studies in the past two decades (Yeung and Chan, 2021). For example, studies using fNIRS found that the activation in the FP of patients with severe depression was enhanced by acupuncture (Tang et al., 2018); The activation in the FP and the DLPFC of patients with trapezius myofascial pain syndrome was suppressed by the electronic wrist-ankle acupuncture (Shi et al., 2022); The functional connectivity within the brain network was increased induced by the acupuncture manipulation (Fernandez Rojas et al., 2019). However, fNIRS was used for the first time to investigate brain functional activities in the bilateral PFC and the bilateral somatosensory cortex during acupuncture manipulations in the current study. The findings of this study verified that fNIRS is a feasible neuroimaging approach to explore the central mechanism of reinforcing-reducing manipulations.

There are still three potential limitations in this preliminary study. Firstly, it’s not sure whether the reinforcing-reducing manipulation has a specific effect on deficiency syndrome and excess syndrome since only healthy subjects were enrolled. Secondly, it’s not clear whether gender influences the reinforcing-reducing manipulation’s effect for that only males were included due to the relatively short length of probes in the fNIRS instrument. Thirdly, the sample size of this pilot study is relatively small. Males and females in pathological state are supposed to be included in the further study with a larger sample size to further investigate acupuncture manipulation’s specific clinical significance and value.

## 5. Conclusion

In conclusion, this study using fNIRS discovered the reinforcing-reducing manipulation induced opposite hemodynamic responses in the bilateral DLPFC and the left S1. The regulation on the DLPFC-S1 may be one of the potential central mechanisms for the realization of the reinforcing-reducing manipulations’ effect. These findings verified the feasibility of utilizing fNIRS to investigate the central mechanism of acupuncture manipulations and provided a new approach to future modern research of traditional acupuncture manipulations.

## Data availability statement

The original contributions presented in this study are included in the article/Supplementary material, further inquiries can be directed to the corresponding authors.

## Ethics statement

The studies involving human participants were reviewed and approved by the Institutional Review Board of the Hospital of Chengdu University of Traditional Chinese Medicine. The patients/participants provided their written informed consent to participate in this study.

## Author contributions

FZ and TY were responsible for this study. FZ, TY, JC, YQ, and LC contributed to the study conception and design. TL and JX participated in participant recruitment. JC and ZT performed the data collection and data analysis. JC wrote the first draft of the manuscript. YG, ZL, and XY provided laboratory-instrument support. JG managed and maintained research data. All authors contributed to the article and approved the submitted version.
