# Supplementary material for: The regulations on cortical activation and functional connectivity of the dorsolateral prefrontal cortex-primary somatosensory cortex elicited by acupuncture with reinforcing-reducing manipulation
Source: Front Hum Neurosci. 2023 May 3;17:1159378. doi: 10.3389/fnhum.2023.1159378 (PMC10188977; doi:10.3389/fnhum.2023.1159378)
Supplement: Supplementary file 1 [file Table_1.DOCX]

# *Supplementary Material*

# The Regulations on Cortical Activation and Functional Connectivity of the DLPFC-S1 Cortex Elicited by Acupuncture with Reinforcing-Reducing Manipulation

**Jingya Cao^1,2,†^, Yuzhu Qu^1,2,†^, Li Chen^1,2,†^, Tianyu Liu^2,3^, Jing Guo^1^, Yulai Gong^4^, Zilei Tian^1,2^, Jing Xiong^5^, Zhenfang Lin^4^, Xin Yang^4,6^, Tao Yin^1,2,*^, Fang Zeng^1,2,*^**

***Correspondence:** Fang Zeng and Tao Yin are corresponding authors

E-mail address: zengfang@cdutcm.edu.cn or [yintao@cdutcm.edu.cn](mailto:yintao@cdutcm.edu.cn)

**Supplementary Table 1.** The MNI coordinates of channels.

| **Channel** | **MNI** | | | **Brain area** | **Brodmann label** |
| --- | --- | --- | --- | --- | --- |
|  | **x** | **y** | **z** |  |  |
| CH1 | -47 | 43 | 24 | DLPFC | BA46 |
| CH2 | -30 | 46 | 42 | DLPFC | BA9 |
| CH3 | -49 | 49 | -1 | DLPFC | BA46 |
| CH4 | -34 | 64 | -9 | OFC | BA11 |
| CH5 | -42 | 55 | 15 | DLPFC | BA46 |
| CH6 | -26 | 68 | 4 | FP | BA10 |
| CH7 | -14 | 68 | 24 | FP | BA10 |
| CH8 | -10 | 46 | 52 | DLPFC | BA9 |
| CH9 | 1 | 55 | 41 | DLPFC | BA9 |
| CH10 | 13 | 46 | 53 | DLPFC | BA9 |
| CH11 | -11 | 73 | -5 | FP | BA10 |
| CH12 | 3 | 69 | 13 | FP | BA10 |
| CH13 | 14 | 73 | -5 | FP | BA10 |
| CH14 | 17 | 68 | 25 | FP | BA10 |
| CH15 | 29 | 69 | 5 | FP | BA10 |
| CH16 | 45 | 55 | 16 | DLPFC | BA46 |
| CH17 | 34 | 45 | 43 | DLPFC | BA9 |
| CH18 | 50 | 43 | 26 | DLPFC | BA46 |
| CH19 | 37 | 65 | -10 | OFC | BA11 |
| CH20 | 52 | 48 | 0 | DLPFC | BA46 |
| CH21 | 57 | 38 | 1 | DLPFC | BA46 |
| CH22 | 65 | -18 | 44 | S1 | BA1 |
| CH23 | 55 | -32 | 58 | S1 | BA1 |
| CH24 | 44 | -16 | 68 | M1 | BA4 |
| CH25 | 70 | -35 | 29 | S2 | BA40 |
| CH26 | 62 | -49 | 46 | S2 | BA40 |
| CH27 | 40 | -48 | 68 | S2 | BA40 |
| CH28 | 29 | -31 | 75 | M1 | BA4 |
| CH29 | -62 | -48 | 45 | S2 | BA40 |
| CH30 | -69 | -34 | 27 | S1 | BA2 |
| CH31 | -29 | -31 | 74 | M1 | BA4 |
| CH32 | -40 | -47 | 68 | S2 | BA40 |
| CH33 | -43 | -16 | 67 | M1 | BA4 |
| CH34 | -54 | -31 | 57 | S1 | BA1 |
| CH35 | -64 | -17 | 42 | S1 | BA1 |
| CH36 | -55 | 38 | -1 | DLPFC | BA46 |

**Note:** BA, Brodmann area; CH, channel; MNI, Montreal Neurological Institute; DLPFC, dorsolateral prefrontal cortex; OFC, orbitofrontal cortex; FP, frontopolar area; S1, primary somatosensory cortex; S2, secondary somatosensory cortex; M1, primary motor cortex.

**Supplementary Table 2.** Cortical activation with significant differences during each reinforcing-reducing manipulation

| **Channel** | **Side** | ***T* value** | ***P*_(FDR)_** |
| --- | --- | --- | --- |
| **LTRei** | | | |
| CH21 | R | 3.43 | 0.0047 |
| CH25 | R | 2.90 | 0.0091 |
| CH30 | L | 2.86 | 0.0067 |
| CH36 | L | 2.80 | 0.0058 |
| **LTRed** | | | |
| CH8 | L | -2.80 | 0.0400 |
| CH21 | R | -4.26 | 0.0018 |
| **ERR** | | | |
| CH9 | L | -2.87 | 0.0036 |
| CH10 | R | -3.28 | 0.0032 |
| CH12 | L | -2.43 | 0.0064 |
| CH17 | R | -2.43 | 0.0058 |
| CH22 | R | -2.06 | 0.0117 |
| CH24 | R | -3.18 | 0.0020 |
| CH25 | R | -2.62 | 0.0055 |
| CH29 | L | -3.22 | 0.0025 |
| CH30 | L | -2.52 | 0.0060 |
| CH34 | L | -3.33 | 0.0057 |

**Note:** CH, channel; LTRei, lifting-thrusting reinforcing manipulation; LTRed, lifting-thrusting reducing manipulation; ERR, even reinforcing-reducing manipulation; R, right; L, left.
